# Efficacy and Safety of the Lateral Abdominal Approach for Lumboperitoneal Shunts in the Post-surgical Abdomen

**DOI:** 10.7759/cureus.77613

**Published:** 2025-01-18

**Authors:** May Pyae Kyaw, Tatsuya Tanaka, Eiichi Suehiro, Takashi Iimori, Takashi Agari, Kazuaki Shimoji, Takashi Sugawara, Hiroshi Itokawa, Keisuke Onoda, Akira Matsuno

**Affiliations:** 1 Department of Neurosurgery, International University of Health and Welfare Narita Hospital, Narita, JPN

**Keywords:** abdominal surgery history, computed tomography, lateral abdominal approach, lumboperitoneal shunt, normal pressure hydrocephalus, peritoneal adhesions, preoperative imaging

## Abstract

Background: Patients with previous abdominal surgeries are often considered unsuitable for lumboperitoneal (LP) or ventriculoperitoneal shunt placement due to the potential risk of peritoneal adhesions. This study evaluates the safety and efficacy of the lateral abdominal approach for LP shunt insertion in such patients.

Methods: A retrospective analysis was performed on 21 patients who underwent LP shunt placement for idiopathic normal pressure hydrocephalus (iNPH) via the lateral abdominal approach at our institution between January 2021 and December 2022. We reviewed patient demographics, surgical outcomes, and preoperative CT imaging to assess for abdominal adhesions and evaluate the procedure's feasibility, ensuring a thorough evaluation of the lateral abdominal approach.

Results: Nineteen patients were included in the final analysis, with a mean age of 72 years (36-85 years). The cohort consisted of 12 male patients and seven female patients, with a mean BMI of 24.3 kg/m². No intraoperative complications or bowel adhesions were observed during catheter placement. Preoperative CT scans showed a mean minimum distance between the peritoneum and bowel of 3.9 mm. No significant differences were found in characteristics or imaging findings between patients with and without a history of abdominal surgery.

Conclusion: The lateral abdominal approach for LP shunt insertion has been shown to be safe and effective, even in patients with previous abdominal surgeries, as it reduces the risk of peritoneal adhesions. However, it is important to note that preoperative CT imaging alone may not be sufficient to predict adhesions. With further research and refinement, the lateral abdominal approach is valuable in treating iNPH.

## Introduction

Patients with a history of abdominal surgery are often deemed unsuitable for ventriculoperitoneal (VP) or lumboperitoneal (LP) shunts due to the potential for peritoneal adhesions [1-6]. Peritoneal adhesions can result in complications such as bowel injury during catheter placement and improper catheter positioning.

The efficacy of LP shunts in managing idiopathic normal pressure hydrocephalus (iNPH) has been well-documented [7]. Among the available techniques, the lateral abdominal approach shows promise for LP shunt insertion [8-10]. This method is particularly advantageous for patients with previous abdominal surgeries, as the insertion site can be strategically located away from prior surgical scars [9,10]. By minimizing the risk of adhesions, this approach provides a safer and more effective alternative for LP shunt placement.

Despite the absence of a standardized preoperative method to evaluate intraperitoneal adhesions, imaging modalities such as ultrasound, CT, and MRI have been investigated for this purpose [11-16]. Postoperative adhesions typically result from the loss of the visceral peritoneum, which promotes adhesion formation between the bowel and the abdominal muscle fat layer [3,11,14]. On CT imaging, indicators of peritoneal and bowel adhesions include the loss of the fat layer behind the abdominal muscle sheath, the absence of the fat layer in the preperitoneal space, and the unchanged bowel position on serial imaging [11,14].

This study aims to evaluate the feasibility of the lateral abdominal approach for LP shunt placement and to assess the diagnostic accuracy of preoperative CT imaging in identifying adhesions between the abdominal wall and bowel.

## Materials and methods

Study population

We retrospectively analyzed the medical records of 21 patients who underwent LP shunt placement via the lateral abdominal approach at Narita Hospital, International University of Health and Welfare, from January 2021 to December 2022. All surgeries were supervised by a single neurosurgeon certified by the Japan Neurosurgical Society.

Methods

Patient demographics, including age, sex, hydrocephalus etiology, history of abdominal surgery, and body mass index (BMI), were collected from medical records. Surgical records and pre-and postoperative abdominal CT images were reviewed. Surgical outcomes were assessed based on the success of abdominal catheter placement and the presence of peritoneal adhesions.

Surgical procedure

**Figure 1:**
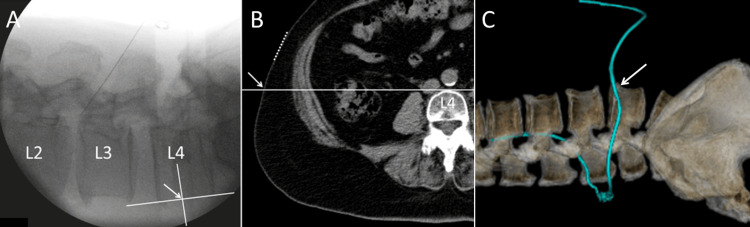
Lateral Abdominal Approach at the Skin Incision Site (A) Fluoroscopic View; (B) Abdominal Computed Tomography; (C) Three-Dimensional Computed Tomography The patient is positioned in the left lateral decubitus position, and a 4 cm skin incision (dotted line) is made 3 cm anterior to the L4 vertebral body level (arrow), guided by fluoroscopy.

The lateral abdominal approach was performed without repositioning the patient (Figure 1). Under general anesthesia, the patient was positioned in the left lateral decubitus position with the spine stabilized to prevent excessive rotation. Using fluoroscopic guidance, a 4 cm skin incision was made 3 cm anterior to the L4 vertebral level. The external oblique, internal oblique, and transversus abdominis muscles were bluntly dissected along their fibers to expose the peritoneum. Peristalsis of the bowel was confirmed through the peritoneum, which was subsequently elevated and incised to access the peritoneal cavity.

CT imaging of the LP shunt insertion site

CT imaging was performed with a slice thickness of 0.5 mm using an Aquilion Prime SP scanner (Canon Medical Systems, Otawara, Japan) with the following parameters: tube voltage 120 kV, tube current CT-AEC (Automatic Exposure), pitch factor 0.813, helical pitch 65, and rotating gantry speed 0.5 s/r. The images were saved in DICOM format.

**Figure 2:**
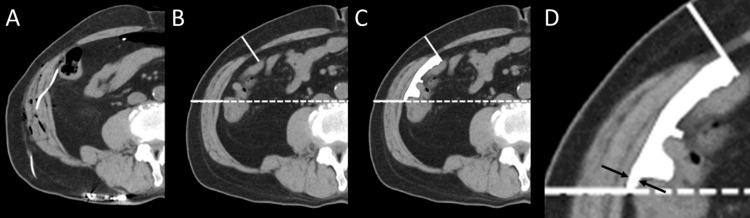
Method for Measuring the Minimum Distance Between the Transversus Abdominis Muscle and Intestine at the Ventral Catheter Insertion Site (A) Postoperative abdominal CT scan showing the ventral catheter insertion site following LP shunt surgery. (B) Preoperative CT scan showing the ventral catheter insertion level. (C) Preoperative CT scan revealing the space (white area in C) between the transversus abdominis muscle and the intestine at the same level. (D) Measurement of the minimum distance between the transversus abdominis muscle and intestine (black arrow). LP: Lumboperitoneal

Initially, postoperative CT scans were used to confirm the placement of the abdominal catheter. Subsequently, preoperative CT scans were reviewed to measure the minimum distance between the transversus abdominis muscle and the intestine at the level where the abdominal catheter was inserted into the peritoneal cavity, as confirmed by the postoperative CT (Figure 2).

Statistical analysis

Continuous variables, such as BMI, age, and the distance between the peritoneum and bowel, were analyzed using the t-test. Binary variables, including sex, were compared using the chi-square test. A p-value of <0.05 was considered statistically significant.

## Results

Patient characteristics

Of the 21 patients initially considered, two were excluded due to the absence of preoperative abdominal CT scans, leaving 19 patients for analysis (Table 1). The mean patient age was 72 years, with a median age of 76 years (36-85 years). The cohort included 12 male patients and seven female patients. The mean BMI was 24.3 kg/m², with a median of 24.0 kg/m² (17.0-31.6 kg/m²). iNPH was the most common etiology, observed in 13 patients. Other causes included brain tumor (one case), post-cerebral hemorrhage (one case), and traumatic brain injury (two cases).

**Table 1:** Characteristics of 19 Patients Who Underwent Lumboperitoneal Shunting Using the Lateral Abdominal Approach

Characteristics	
Age at surgery, median years(range)	76(36-85)
Male, n (%)	12(63.2%)
Body mass index, median kg/m^2^ (range)	24.0(17.0-31.6)
History of prior abdominal surgery, n (%)	7(36.8%)
Intraoperative findings of intestinal adhesions, n (%)	0(0%)
Distance between transversus abdominis and intestine at the abdominal catheter entry site, median mm (range)	2.2(0-20.4)

Seven patients had a history of abdominal surgery, all of which were open surgeries. These included one case of renal cancer, four cases of appendicitis, one case involving both colon cancer and uterine fibroids, and one case of gastric cancer with concomitant cholecystectomy. In the case with a history of renal cancer, the surgery was performed in a right lateral decubitus position with a left-sided lateral abdominal approach. A left lateral decubitus position and right lateral abdominal approach were used in all other cases.

Outcomes

The ventral catheter was successfully inserted via the lateral abdominal approach without intraoperative complications, and no bowel adhesions were observed.

Preoperative CT scans revealed a mean minimum distance of 3.9 mm between the peritoneum and bowel under the transversus abdominis muscle, with a median of 2.2 mm (0-20.4 mm).

Comparison based on the history of abdominal surgery

Patients with a History of Abdominal Surgery

The mean age in this group was 77 years, with a median of 77 years (75-78 years). This subgroup included four male patients and three female patients. The mean BMI was 23.6 kg/m², with a median of 23.0 kg/m² (17.0-30.9 kg/m²). Preoperative CT findings showed that the mean minimum distance between the peritoneum and bowel under the transversus abdominis muscle was 2.3 mm, with a median of 1.5 mm (0-6.5 mm).

Patients without a History of Abdominal Surgery

The mean age in this group was 70 years, with a median of 73 years (36-85 years). This group consisted of eight male patients and four female patients. The mean BMI was 25.6 kg/m², with a median of 24.6 kg/m² (19.6-31.6 kg/m²). On preoperative CT, the mean minimum distance between the peritoneum and bowel in the flank approach was 4.6 mm, with a median of 2.2 mm (0-13.2 mm).

Comparison of results

The two groups had no significant differences regarding demographic or baseline characteristics (Table 2). Furthermore, no statistically significant differences were observed in the minimum distance between the peritoneum and bowel under the transversus abdominis muscle, as assessed by preoperative CT scans, between patients with and without a history of abdominal surgery.

**Table 2:** Comparison of Patients With and Without Prior Abdominal Surgery

	No prior abdominal surgery n=12	History of prior abdominal surgery n=7	t-value/x^2^-value	p-value
Age at surgery, median years (range)	73(36-85)	77(75-78)	t-value:1.345	0.182
Male, n (%)	8(66.7%)	4(57.1%)	x^2^-value:0.0	1.00
Body mass index, median kg/m^2^ (range)	24.6(19.6-31.6)	23.0(17.0-30.9)	t-value:1.305	0.308
Intraoperative findings of intestinal adhesion, n (%)	0(0%)	0(0%)	x^2^-value:0.0	1.00
Distance between transversus abdominis and intestine at the abdominal catheter entry site	2.2(0-20.4)	1.5(0-6.5)	t-value0.926	0.377

## Discussion

The lateral abdominal approach, while carrying the risk of accidental entry into the retroperitoneal space, offers a strategic advantage for patients with a history of abdominal surgery [8-10]. This approach allows the placement of abdominal catheter insertion sites away from previous surgical scars [9,10]. In this study, despite a history of abdominal surgery, no adhesions between the abdominal wall and intestines were detected using the lateral abdominal approach. This suggests that the lateral approach may enable the safe insertion of abdominal catheters away from previous surgical scars. However, predicting adhesions preoperatively using simple CT scans remains challenging. Although we hypothesized that measuring the thickness of the lateral abdominal muscle fat layer could help identify adhesions, the results were not statistically significant.

Previous studies have reported varying diagnostic accuracies for detecting adhesions, with ultrasound (US) showing 76-100%, magnetic resonance imaging (MRI) 79-90%, and CT 66% [3,11-13,16]. The sensitivity ranges were 21-100% for US, 22-93% for MRI, and 61% for CT, while specificity ranged from 32 to 100% for US, 25 to 93% for MRI, and 63% for CT [3,11-13,16]. Notably, CT diagnosis of abdominal adhesions using artificial pneumoperitoneum demonstrated a sensitivity of 100%, specificity of 95.04%, and accuracy of 95.46% [12]. Findings suggestive of peritoneal and intestinal adhesions on CT include the loss of the fat layer behind the anterior abdominal muscle sheath and preperitoneal space, unchanged bowel positioning on serial scans, and localized peritoneal thickening and enhancement [11,14].

MRI is considered a highly accurate diagnostic modality capable of visualizing adhesions between organs. However, it tends to overestimate the presence of adhesions [3,11]. On the other hand, CT currently lacks sufficient evidence to be deemed reliable for diagnosing adhesions [11,14]. Abdominal US is widely used to assess adhesions between the intestines and the abdominal wall [11,13,15,16], and the visceral slide technique enables non-specialist surgeons to quickly and accurately identify areas free of adhesions [16].

Postoperative adhesions often occur due to the loss of the visceral peritoneum, leading to adhesion formation between the intestines and the anterior rectus muscle fat layer [3,11,14]. Such adhesions can restrict bowel movement, causing the bowel to become "fixed," which may be confirmed through serial CT or MRI imaging [3,11,12,14]. However, in this study, the fixation of the retroperitoneally located ascending colon did not indicate adhesions. Even in patients with a history of abdominal surgery, no adhesions were identified between the peritoneum and intestines via the lateral abdominal approach.

In patients undergoing VP shunt placement, the catheter is typically positioned on the right side to avoid complications associated with the dominant hemisphere [17]. In contrast, LP shunt allows for catheter placement on either side. For patients with a history of appendicitis, particularly those who experienced severe peritonitis, inflammation on the right side can lead to adhesions. In such scenarios, left-sided abdominal catheter insertion in the right lateral decubitus position is recommended. Additionally, LP shunt may be preferable to VP shunt when avoiding the side of prior abdominal surgeries. In this study, we report a patient with a history of right renal cancer surgery who successfully underwent left lateral abdominal catheter placement in the right lateral decubitus position.

This study has several limitations. First, the relatively small sample size may limit the generalizability of the findings. A larger cohort is necessary to confirm whether the lateral abdominal approach is consistently safe for catheter insertion in patients with a history of abdominal surgery. Second, the diagnostic accuracy of preoperative imaging, particularly CT scans, for predicting adhesions remains limited. Although we measured the distance between the abdominal wall and intestines to estimate adhesions, the results were not statistically significant. Advanced imaging modalities or novel techniques may be needed to improve preoperative adhesion diagnosis. Lastly, the absence of a control group undergoing traditional catheter placement methods limits the ability to directly compare outcomes. Future randomized controlled trials comparing the lateral abdominal approach with conventional techniques are warranted to validate the observed benefits.

## Conclusions

In conclusion, the lateral abdominal approach for LP shunts appears to be safe and effective, even in patients with a history of abdominal surgery. Importantly, no adhesions between the peritoneum and intestines were observed during the procedure. However, predicting adhesions preoperatively based solely on CT imaging remains difficult, underscoring the need for more reliable diagnostic tools. Future studies are warranted to confirm these findings and further enhance preoperative adhesion evaluation techniques.
